# Agronomic biofortification of food crops: An emerging opportunity for global food and nutritional security

**DOI:** 10.3389/fpls.2022.1055278

**Published:** 2022-12-09

**Authors:** Ajay Kumar Bhardwaj, Sukirtee Chejara, Kapil Malik, Raj Kumar, Ashwani Kumar, Rajender Kumar Yadav

**Affiliations:** Central Soil Salinity Research Institute, Karnal, Haryana, India

**Keywords:** biofortification, micronutrient, malnutrition, nanofertilizers, food and nutritional security (FNS)

## Abstract

Fortification of food with mineral micronutrients and micronutrient supplementation occupied the center stage during the two-year-long Corona Pandemic, highlighting the urgent need to focus on micronutrition. Focus has also been intensified on the biofortification (natural assimilation) of mineral micronutrients into food crops using various techniques like agronomic, genetic, or transgenic. Agronomic biofortification is a time-tested method and has been found useful in the fortification of several nutrients in several crops, yet the nutrient use and uptake efficiency of crops has been noted to vary due to different growing conditions like soil type, crop management, fertilizer type, etc. Agronomic biofortification can be an important tool in achieving nutritional security and its importance has recently increased because of climate change related issues, and pandemics such as COVID-19. The introduction of high specialty fertilizers like nano-fertilizers, chelated fertilizers, and water-soluble fertilizers that have high nutrient uptake efficiency and better nutrient translocation to the consumable parts of a crop plant has further improved the effectiveness of agronomic biofortification. Several new agronomic biofortification techniques like nutripriming, foliar application, soilless activation, and mechanized application techniques have further increased the relevance of agronomic biofortification. These new technological advances, along with an increased realization of mineral micronutrient nutrition have reinforced the relevance of agronomic biofortification for global food and nutritional security. The review highlights the advances made in the field of agronomic biofortification *via* the improved new fertilizer forms, and the emerging techniques that achieve better micronutrient use efficiency of crop plants.

## 1 Introduction

**Figure 1:**
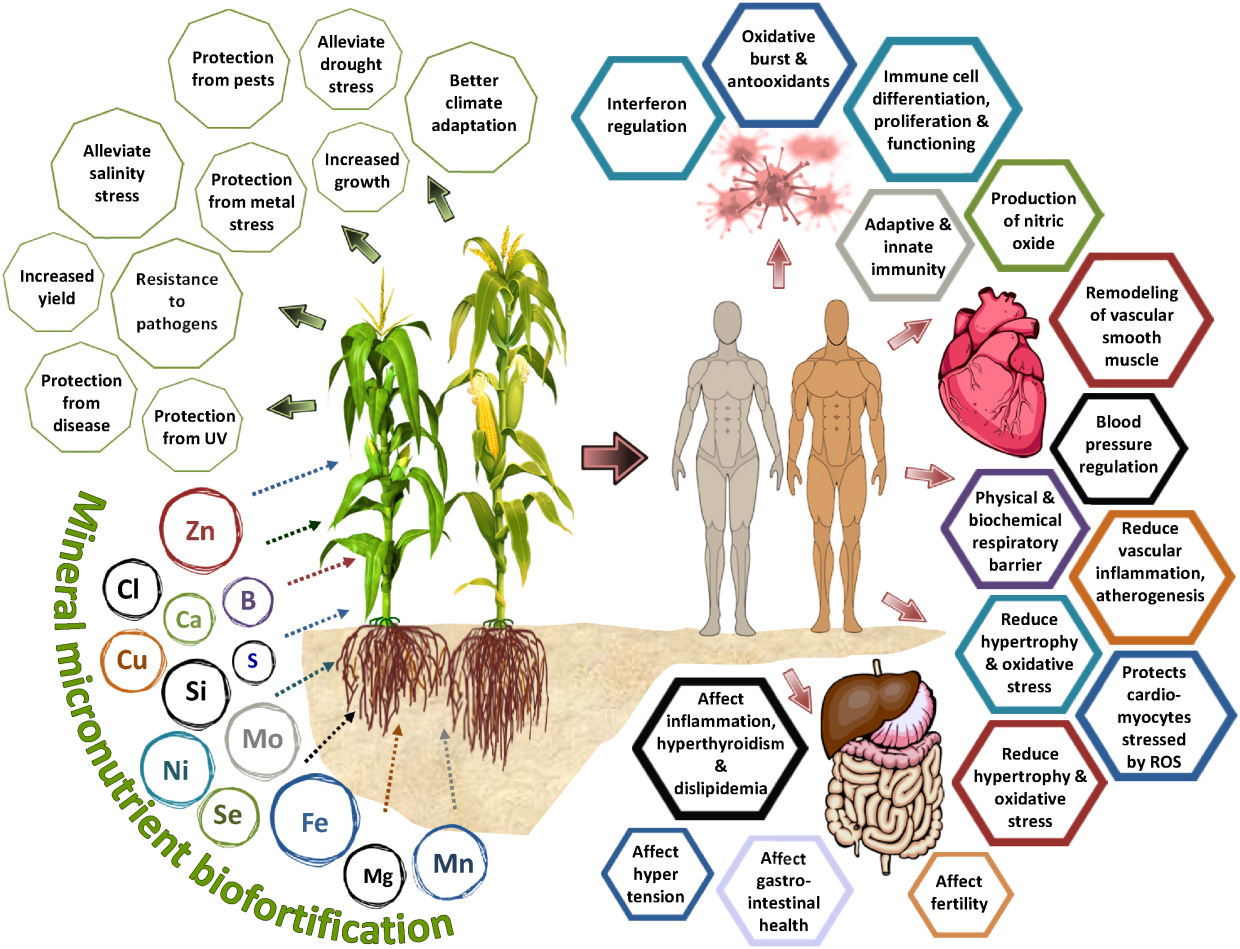
Influence of mineral micronutrient biofortification on the plant physiological processes and its relation to human health and immunity.

Around the globe, two billion people face micronutrient deficiency and acute malnutrition, mainly pregnant women, and children under the age of five (White and Broadley, 2009; WHO, 2012). More people are affected by the lack of micronutrients than the issue of low energy intake and poor dietary quality (Stewart et al., 2010). The deficiency of vitamin A, zinc, iron and iodine causes the death of around 20% of children under the age of five (Prentice et al., 2008). Cereal-based foods represent the major dietary habit of micronutrient-deficient populations (Cakmak, 2010; Bouis et al., 2011). Estimates suggest that 149.2 million children under the age of five are stunted, with 45.4 million reported “wasted,” weight that is out of proportion to their height. Malnutrition is responsible for almost 45 percent of fatalities among children under the age of five (World Health Statistics, 2022). The situation is significantly worse in regions like Southeast Asia where 30.1 percent and 14.5 percent of children under the age of five are stunted and wasted, respectively, compared to 22 percent and 6.7 percent globally (World Health Statistics, 2022). Several factors associate with malnutrition but the most emphasized is the lack of a balanced diet. According to the global nutrition report (2017), poor nutrition causes an 11 percent loss of gross domestic product in Asia and Africa. In Africa, East Asia, and the Pacific, the Hidden Hunger Index (HHI), a measure of malnutrition, has shown a downward trend. Though malnutrition is mainly brought on by a lack of consumption of fruits, vegetables, food derived from animals, and nutrient-rich foods, many a world’s poor cannot afford these foods and mainly rely on cereals and reasonably priced staples. This improvement was obtained by simply increasing Zn and vitamin A intake (Ruel-Bergeron et al., 2015), implying that excellent effects can be expected if other micronutrients are also considered. Though the recent focus has been on micronutrient supplements and industrial fortification of foods to target vulnerable segments of society, the long-term and sustainable impacts can only be achieved by micronutrient fortification at the crop production stage *via* their natural assimilation by plants (**Figure 1**). Fortification is the enhancement of nutrient density in food through physical interventions such as the addition of salts while biofortification refers to enhancing the levels of bioavailable micronutrient using techniques such as conventional plant breeding, transgenics, and agronomic biofortification (i.e., use of micronutrient-rich fertilizers) (FAO, 2017). Until now, the primary focus of crop production has been on increasing crop yields and agriculture productivity rather than human nutrition and health. This approach has gradually led to micronutrient malnutrition, worldwide (Development initiatives, 2017). Besides, climate change impacts on crop productivity, unbalanced fertilization, and degradation of soil quality have impacted the quality of crop based food available to poor people, worldwide. Since maximum yield benefits come from the use of macronutrient (nitrogen, phosphorus, and potassium) fertilizers, the use of micronutrient fertilizers is also below recommended in many parts of the world. The skewed fertilization has created environmental problems besides malnutrition. Biofortification of cereal and staple crops offers a sustainable solution to meet the micronutrient demand of the human body to maintain better health.

From an economic point of view, industrial fortification of processed foods seems a costly approach with only marginal benefits of fortification for micronutrients like Fe and Zn (Bromage et al., 2018). Biofortification can address this issue yet the ideal way to promote biofortification would be as a part of a large portfolio of environmentally friendly, food-based nutritional strategies (FAO, 2017). Furthermore, population explosion is an expected concern in the near future along with predicted climate change. Achieving food and nutritional security under such conditions would be a major challenge. Keeping this in view, international organizations such as the World Health Organization (WHO) and Consultative Group on International Agricultural Research (CGIAR) have included the development of nutritionally- enhanced biofortified crops as one of their main goals. Agronomic biofortification is the oldest and most widely adopted technique so far, yet it seems to be losing its importance to the biotechnological and breeding approaches. In this context, this review compiles and discusses its advantages and progress in relation to human nutrition and health, the challenges that it faces, and technological advances that would enhance its future relevance. The review further described the advances made in the field of the improved new fertilizer forms that can enhance agronomic biofortification efficiency, and the emerging techniques that achieve better micronutrient uptake by the crop plants.

## 2 Mineral micronutrients in context to human health and nutrition

Human nutrition largely depends on plants, directly or indirectly. Micronutrient deficit in plants can lead to micronutrient deficiency in people who eat those plants and their processed products as a source of nutrition (Joy et al., 2015; Oliver and Gregory, 2015). The countries where staple foods are grains and tubers cultivated on nutrient-depleted soils suffer from widespread Zn deficiency, which leads to stunting and child death (Cakmak, 2009; White and Brown, 2010; Joy et al., 2015). As a result, micronutrient fertilization (agronomic biofortification) may be needed to address both crop nutritional quality and human dietary micronutrient requirements. Poor people, who are deprived of fresh fruits and vegetables have very little intake of nutrients because of complex social and economic circumstances (Shenkin, 2006). Particularly, children and women would benefit the most from a micronutrient supplement if biofortified crops are included in their daily diet. Zinc, one of the many micronutrient elements essential for good health, is frequently low in the human diet. Zn deficiency can impair immunological function, restrict children’s growth, and harm women’s pregnancy outcomes (Hess and King, 2009). Similarly, a lack of Fe in the diet leads to a variety of physiological problems such as anemia and neurological illnesses (Andreini et al., 2006). Weingartner (2005) argues that food security is just as crucial as food security. Improving the nutritional quality of agricultural products could be the most effective and long-term method to ensure food security (Cakmak, 2008; Hotz, 2009; Gomez-Galera et al., 2010). The Biofortification of crops and enhancing the bioavailability of nutrients in the edible component of the crop can help to prevent micronutrient deficiency. Biofortification of crops, whether agronomic or genetic, can be achieved with a little additional cost that is significantly less than the risk of hunger and malnutrition.

Under the prevailing condition of the Covid-19 pandemic, micronutrient supplementation occupied center stage for providing an important role in providing resistance to respiratory virus infection (Calder, 2020). Micronutrients support and influence each stage of an immune response. Micronutrient malnutrition can affect both innate and adaptive immunity, causing immune suppression and hence increasing susceptibility to infection (Gorji and Ghadiri, 2021). Inadequate nutritional status and infections have a synergistic relationship. An infection aggravates the nutritional deficiency status of the body and causes increased micronutrient demand (Al Sayah et al., 2021). Viral infections are a major cause of morbidity and mortality throughout the world (Erdem and Unal, 2015), as demonstrated by seasonal influenza and the outbreak of the novel coronavirus (COVID-19). Zinc being an essential micronutrient modulates the function of approximately 2000 enzymes and 750 transcription factors involved in different metabolic processes including immune response (Carr and Maggini, 2017; Read et al., 2019; Chasapis et al., 2020). Zinc also possesses a variety of antibacterial properties such as inhibition of RNA-dependant RNA polymerase enzyme that promotes replication of SARS-CoV-2 by pyrrolidine dithiocarbamate; a Zn ionophore, was found responsible for this inhibition (Suara and Crowe Jr, 2004; Ghaffari et al., 2019; Read et al., 2019). As the Zn cofactor functions in metalloenzyme, it also helps in maintaining the integrity of immune barriers (Lin et al., 2017; Chasapis et al., 2020). The cytotoxic nature of natural killer cells and cellular function, growth, and differentiation of innate immune cells is also influenced by the activity of Zn (Sheikh et al., 2010; Gao et al., 2018; Maggini et al., 2018). It also has anti-inflammatory and antioxidant properties through the modulation of cytokine release and antioxidant proteins (Wintergerst et al., 2006; Maggini et al., 2008). Zinc promotes the proliferation of cytotoxic T-Cells and is also involved in antibody production, mainly immunoglobulin G antibodies (Rink and Kirchner, 2000; Maggini et al., 2008). The deficiency of Zn increases inflammatory disorder and viral pneumonia in the elderly and children (Caplan et al., 2007; Maggini et al., 2008; Savino and Dardenne, 2010). Zinc supplementation in children reduces susceptibility and severity as well as the duration of pneumonia and the common cold (Prentice, 2017; Read et al., 2019). In the elderly, its supplementation can increase the serum Zn level and number of T-cells (Barnett et al., 2016). Its supplementation can also benefit the management of COVID-19, as high risk prevails under Zn deficiency. Copper can decrease inflammatory markers and can prevent oxidative DNA damage. It is also a component of the Zn-Cu-superoxide dismutase antioxidant enzyme (Hemila, 2017). Due to its function in T-cell proliferation and natural killer activity, copper deficiency is linked to an increased rate of infection (Beyhan-Sagmen et al., 2017). Iron is important for epithelial tissue development and growth, as well as for the formation of reactive oxygen species that combat infections (Inoue et al., 1998). Intake of iron has been shown to help fight respiratory infections (Kim et al., 2016) pulmonary iron regulation is thought to be a defense mechanism against respiratory pathogens (Hemila and Chalker, 2019). Aside from that, trials in several nations have shown that Zn, in combination with vitamin A, can be a powerful weapon against diarrhea and pneumonia, two of the most common childhood illnesses (Bhargava et al., 2001).

Both prokaryotic and eukaryotic cells require iron to perform their essential tasks. Iron is required for the activity of numerous proteins, including as haemoglobin (Hb), and enzymes that work with DNA and RNA, such as ribonucleotide reductase and DNA primase (Drakesmith and Prentice, 2008). Additional indicators for determining iron status include Hb, mean cell volume, mean cell haemoglobin, serum ferritin, soluble transferrin receptors, transferrin saturation, and total iron-binding capacity (Northrop-Clewes, 2008). A lack of iron makes people more susceptible to viral infections, and iron regulation is important for the host cell’s defense mechanism (Tarifeño-Saldivia et al., 2018). Aging is a result of an imbalance between the body’s antioxidative defenses and the damage reactive oxygen species (ROS) cause. The nutritionally significant trace element selenium (Se) may repair gradual and spontaneous physiological changes brought on by oxidative stress, potentially preventing disease and fostering healthy aging. Se strengthens the immune system, the metabolic balance, and the antioxidant defense system. Low Se status might reduce life expectancy by accelerating aging and increasing susceptibility to diseases including cancer and immune system issues (Bjorklund et al., 2022). Since selenium (Se) has beneficial antioxidant effects, it has been used as a dietary supplement for enhancing health. Because it is involved in enhancing antioxidant defense, immunological functions, and metabolic homeostasis, it may remodel gradual and spontaneous biochemical and physiological changes that could result in disease prevention and healthy aging (Malavolta and Mocchegiani, 2018). For essential copper-dependent enzymes, which are encoded by both prokaryotes and eukaryotes, most organisms need copper as a cofactor. Copper is an essential micronutrient. Copper is a crucial component of the immune system in mammals. Phagocytic cells exploit the antibacterial toxicity of copper, which accumulates in infection locations such as the gastrointestinal and respiratory tracts, blood, and urine, to directly kill germs. Diets high in copper make people less susceptible to illness than diets deficient in copper (Focarelli et al., 2022). Iodine has been proposed as a potential treatment for COVID-19 infection and to lessen the side effects of vaccination. Iodine is used by the thyroid gland to make thyroid hormones. In addition to being a component of thyroid hormones, iodine serves as an antioxidant, anti-inflammatory, anti-proliferative, and differentiation agent. Iodine helps to maintain the health of organs that can absorb it by having effects that are mediated by a variety of various processes or pathways that have either direct or indirect activities (Boretti and Banik, 2022).

## 3 The science of biofortification in brief

Higher micronutrient intake, improved translocation within the plant, and increased accumulation in the edible portions are all required for optimal biofortification. There are two significant concerns with plant-based micronutrient supply. The first is that plants cannot synthesize (or adequately take up) many of the minerals essential for human survival, and the second is that these nutrients are distributed and concentrated unevenly in different plant sections (Zhu et al., 2007). Plant components such as leaves, stems, and roots, for example, have far greater Fe concentrations than rice grains. Bioavailability and absorption of these nutrients in the human gut are also essential, although these topics are outside the scope of this review. To better understand and attain mineral micronutrient concentration and bioavailability, the following sections provide a summary of physiological mechanisms behind biofortification, and methodologies utilized to achieve better biofortification.

### 3.1 Physiological processes in nutrient transport and biofortification

Root uptake followed by xylem loading is the primary step in the process of nutrient acquisition and accumulation in plants. Thus, the knowledge of the forms in which the micronutrients are available in the soil and taken up by plants along with the supply and limitations of micronutrients are important to consider (Bhardwaj et al., 2022; White and Broadly, 2009). Plants do not use the same strategy to take up all the nutrients. For instance, in non-graminaceous and dicot species, Fe acquisition is based on the principle of Fe limiting environment. In this condition, an active proton pump increases the solubility of Fe^+3^, *via* a ferric chelate reductase to generate more Fe^+2^ and a Fe transporter. Whereas in graminaceous monocots, the strategy is based on the release of phytosiderophores from roots which chelate with Fe^+3^ and take up this complex by specific transporters (Hell and Stephan, 2003). Some transporters are associated with Zn (Assuncao et al., 2010) and Cu (Delpozo et al., 2010) uptake. Transporters of ZIP family (*Z*RT-IRT-like *P*roteins named after the high-affinity yeast plasma membrane Zn uptake transporter, ZRT1, and the high-affinity *Arabidopsis thaliana* plasma membrane Fe uptake transporter, IRT1) transport several micronutrients including Zn, Cu, Mn, and Cd (Eckhardt et al., 2011). Increased flux of nutrients into the shoot indirectly activates homeostasis that increases root uptake levels and possible molecular targets include FRD3, HMA2, MHA4, HMA5, and MTP3. Once the micronutrients enter the xylem, transpirational forces pull the nutrients upwards and deposit them in the leaves of a plant. Until this stage, the leaves work as a sink for nutrients and carbohydrates.

During the further growth of a plant, leaves transform themselves from a sink (carbon and nutrient importer) to a source (carbon and nutrient exporter), and this transition takes place when the carbon storage through photosynthesis is greater than the requirement of respiration and growth. The transport of nutrients from roots to leaves is *via* the xylem whereas further translocation from leaves to other parts (e.g. fruit, grain, etc.) is *via* the phloem. Xylem unloading and phloem loading are not very well characterized hence they act as a constraint in understanding the translocation of nutrients from leaves to grains or other edible portions of a plant. Nutrients such as Se and Mg are transported quickly in the phloem while other nutrients like Fe, Zn, Cu, Ca and I have little phloem mobility (White and Broadley, 2003). Due to this, phloem-fed tissues like fruits, grains, and tubers are a poor source of nutrients as compared to leafy vegetables in which nutrients are translocated through the xylem (White and Broadley, 2009). Also, in the case of foliar fertilizer application (spray) micronutrients will enter through cuticle and stomata present on the leaf surface and directly accumulate in the phloem; hence this pathway would be shorter than root uptake and this mode of application can be more efficient from biofortification point of view. In the phloem stream, Fe and Zn move in the chelated forms (Blindauer and Schmid, 2010). Major Fe transporters here are ITP (iron transport protein), which was first identified in the phloem of *Ricinus communis* (Kruger et al., 2002), and Nicotinamide (NA), which is present in both dicot and monocots. Besides Fe, NA can also bind with Mn, Zn, Co, Cu, and Ni (Sancenon et al., 2003). The gene responsible for the transport of NA belongs to Yellow Strip Like family (YSL) transporters.

Another significant step is the nutrient deposition in the grains. Grains are connected *via* a single vascular bundle to the maternal plant, and the vascular bundle carries the nutrients to the seed coat (Zhang et al., 2007) and the developing endosperm. Different genes MHA, ZIP, Nramp, NAS, and YSL are involved in this process (Tauris et al., 2009). Fe and Zn are mostly stored in the embryo and aleurone layer while endosperm usually remains poor in terms of micronutrient content. Fe is mainly transported to and located in seed vacuoles by VIT1 protein (Kim et al., 2006). Ferritins in the leaves and globulins, albumins, and glutelins in the seed are important storage spaces for nutrients. Promoter substances and antinutrients also play an important role. Increasing the promoter substances and decreasing the antinutrients can be of immense help in biofortification. Promoter substances such as Vitamin E, Vitamin D, Choline, Niacin, and provitamin A can promote the absorption of Se, Ca, P, Fe, and Zn. Antinutrients are mainly phytate, polyphenols, and oxalate. These are generally indigestible compounds and most deleterious in the process of micronutrient absorption.

### 3.2 Biofortification types

**Figure 2:**
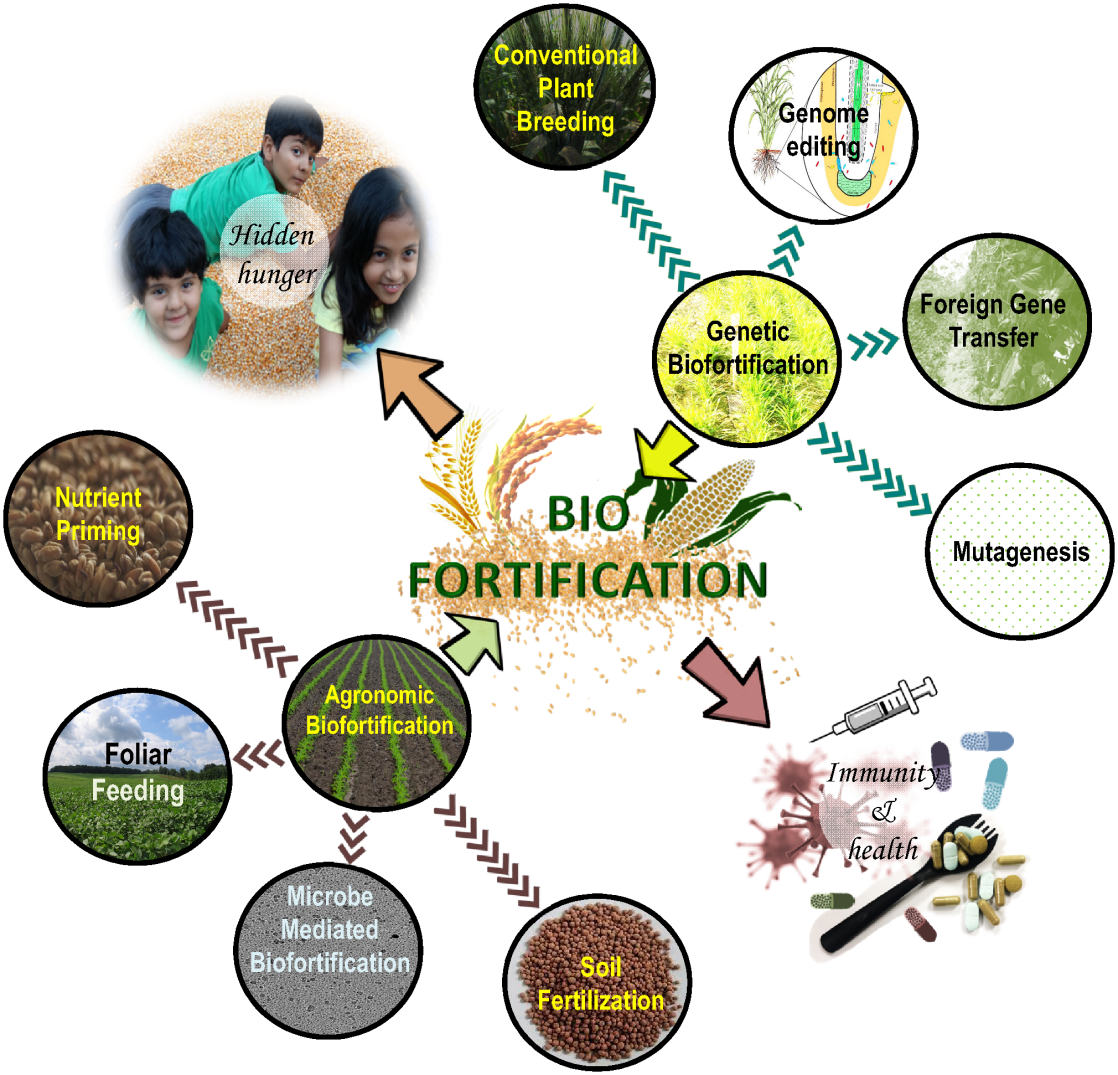
Conventional and advanced biofortification techniques used in crop production and improvement programs.

There are several types of biofortification techniques but broadly the approaches micro can be categorized into two broad categories, one which employs agronomic approaches (e.g. use of micronutrient-rich fertilizers) and the other that employs a genetic breading approach (**Figure 2**). The following sections discuss the most common methodologies adopted so far.

#### 3.2.1 Biofortification through conventional breeding

The conventional breeding method is widely adopted and has gained increased importance during the last decade. In recent years, it has been established as a cost-effective, feasible, and largely approved technique (compared to transgenics) for the fortification of micronutrients (Van Der Straeten et al., 2020). The presence of adequate genotypic variation in the trait of interest is a requirement for using this technique. These already existing variants can be exploited to boost mineral and vitamin levels in crops. Over several generations, superior-quality parent lines with high nutrient content are crossed with recipient lines with other desirable agronomical qualities to generate plants with desired nutrient levels and agronomic traits. The lack of genetic heterogeneity in the gene pool can be a major stumbling block for this strategy; it can be solved by crossing with distant relatives, but it slows the transfer of favorable traits into the intended commercial cultivar. Alternatively, mutagens can be used to create commercial variants. Because breeding is a quick way to improve plants, various organizations throughout the world have started breeding initiatives to improve the micronutrient content of crops.

The Health Gain Initiative (2005-2010), a £10 million project comprising 44 partners from 15 European Union nations, was taken up to enhance food quality. HarvestPlus is a breeding program for biofortified staple food crops created by the CGIAR, the International Center for Tropical Agriculture (CIAT), and the International Food Policy Research Institute (IFPRI). HarvestPlus’ principal purpose is to develop vitamin-A, zinc, and iron-rich staple crops in Asia and Africa (wheat, rice, maize, cassava, pearl millet, beans, sweet potato, and so on) (Bouis and Welch, 2010). The micronutrient status of targeted groups, primarily resource-poor individuals in developing nations, would be significantly improved if the concentration of bioavailable critical minerals and vitamins is increased.

#### 3.2.2 Biofortification through transgenic means

Under the condition of limited or no genetic variation, the transgenic approach can serve as an alternative way for the development of biofortified crops (Brinch-Pedersen et al., 2007). The limitless genetic pool is available in this strategy for the transfer and expression of beneficial genes from any other plant species, regardless of their evolutionary or taxonomic rank. When a crop’s natural ability to absorb a nutrient is inadequate, the transgenic technique appears to be the most viable alternative for fortifying the crop (Perez-Massot et al., 2013). The key to the development of transgenic crops is the identification of gene functions and the use of these genes to alter plant metabolism (Newell-McGloughlin, 2008). Pathways from many organisms, primarily bacteria, can be introduced into crops to discover new metabolic engineering pathways (Newell-McGloughlin, 2008). Simultaneously gene integration can be utilized to improve micronutrient concentrations as well as bioavailability and minimize anti-nutrients that reduce nutrient bioavailability in the plant system.

Genetic manipulation can even shift micronutrients between tissues, improving micronutrient availability in commercial crop edible tissue and thereby increasing the biochemical pathway’s efficiency in edible tissues (Yang et al., 2002; Agrawal et al., 2005). Developing a transgenic crop takes a lot of time, effort, and money during the research and development stage, but if successful, it can be cost-effective and sustainable in the long run. Furthermore, there are no taxonomic restrictions with the transgenic technique, and even artificially generated genes can be used. Reduced micronutrient deficiency among its users, especially poor people in developing nations who cannot afford vitamin supplements, is ensured by increased micronutrient content (Hirschi, 2009). Different crops have been genetically modified to increase their micronutrient content. Vitamins, minerals, essential amino acids, and essential fatty acids are among the micronutrients that have been targeted by utilizing multiple genes to boost nutritional levels in crops. Biofortification targets include ferritin and nicotinamide synthase for mineral nutrients, lycopene-cyclase for vitamins, albumin for vital amino acids, and 6 desaturases for essential fatty acids. High lysine maize, high unsaturated fatty acid soybean, high provitamin A and iron-rich cassava, and provitamin A-rich golden rice are all successful examples of transgenic crops (Hirschi, 2009). Cereals, legumes, vegetables, oilseeds, fodder, and fruit crops containing transgenic biofortification are widely reported.

#### 3.2.3 Biofortification using agronomic methods

Agronomic biofortification is done using micronutrient-enriched fertilizers, and it is a simple and quick measure to increase the nutritional status of the crop, and consumption of such crops improves human nutrition status (Cakmak and Kutman, 2017). Agronomic Biofortification generally relies on methods of fertilizer application, mineral element solubilization, and mobilization from source to sink (consumable parts of a plant). Nitrogen (N), phosphorus (P), and potassium (K) being macro minerals contribute toward higher yield goals. The increased drive to produce and use macronutrient fertilizers during the 1960s led to an immense increase in crop productivity and resulted in the green revolution which saved the world, particularly the developing countries, from starvation. In the present scenario, with a higher yield to feed around seven billion people (Graham et al., 2007) focus is not only on producing more from limited resources but also to enrich consumable parts of the plant with micronutrients for good health. Micronutrients are found to varying degrees in different plant parts and are usually absorbed from the soil. The application of micronutrients as fertilizers can improve micronutrient status in the soil as well as correct their deficiency in plants and humans. Yet in many cases, micronutrients applied to the soil get immediately fixed and do not get readily translocated to the consumable plant parts. Application of micronutrients using other means such as foliar sprays of soluble form is recommended then. If sufficient attention is given to some aspects, such as the fertilizer form, application method, and time of application, agronomic biofortification is a simple and inexpensive tool. Agronomic biofortification using mineral fertilizers is feasible and can be exemplified by the success of Zn fertilization in Turkey (Cakmak, 2009), Se fertilization in Finland (Aro et al., 1995), I fertilization in China (Prom-U-Mai et al., 2020). Agronomic biofortification has proved successful in many crops as detailed in **Table 1**. The key advantage of agronomic biofortification over genetic biofortification is that the fertilizer forms and application techniques are crop non-specific. The fertilizer application rates and their mode of application can be quickly adapted from one crop to another while genetic and transgenic biofortification methods are crop-specific, and therefore bringing more crops into the biofortified profile is highly time-consuming and resource exhaustive.

**Table 1:** Application techniques and the fortification levels achieved for different mineral micronutrients for different crops.

Application technique	Crop	Nutrient	Biofortification level (%)	Reference
**Soil application**	Fodder	Zn	13	Grujcic et al., 2021
		Se	6	Grujcic et al., 2021
	Rice	Zn	7	Joy et al., 2015
			12	Tariq et al., 2007
		Mn	18-28	Swarup et al, 1981
	Wheat	Zn	19	Joy et al., 2015
			11-29	Dwivedi and Srivastva, 2014
			21-31	Shivay et al., 2008
			144-195	Wang et al., 2015
		Mn	17-40	Nayyar et al., 1985
		Zn	32	Chattha et al., 2017
	Corn	Zn	27	Joy et al., 2015
			75	Saleem et al., 2016
		Fe	66	Saleem et al., 2016
	Sorghum	Fe	5-12	Singh et al., 2016
		Zn	5-8	Singh et al., 2016
	Finger millet	Zn	15	Yamunarani et al., 2016
	Chickpea	Zn	6	Hidoto et al., 2016
		Fe	202	Khalid et al., 2015
**Foliar application**	Rice	Zn	31	Prom-U-Thai et al., 2020
			25	Joy et al., 2015
			35	Ram et al., 2021
			36	Naeem et al., 2022
		Fe	8	Prom-U-Thai et al., 2020
		Se	300	Prom-U-Thai et al., 2020
			1307	Naeem et al., 2022
		I	1754	Prom-U-Thai et al., 2020
			1450	Naeem et al., 2022
	Wheat	Zn,	21	Aziz et al., 2019
			99	Pahlavan-Rad and Pessarakli, 2009
			63	Joy et al., 2015
			3-47	Narwal et al., 2012
		B	25	Aziz et al., 2019
		Fe	22	Aziz et al., 2019
			8	Pahlavan-Rad and Pessarakli, 2009
		Fe	6-85	Narwal et al., 2012
		Mn	22	Aziz et al., 2019
			7	Pahlavan-Rad and Pessarakli, 2009
			17-33	Narwal et al., 2012
		Cu	47	Aziz et al., 2019
		Zn	100	Ram et al., 2021
	Corn	Zn	30	Joy et al., 2015
			55	Saleem et al., 2016
		Fe	52	Saleem et al., 2016
	Egg plant	Cu	59	Bana et al., 2021
		Fe	49	Bana et al., 2021
		Mn	47	Bana et al., 2021
		Zn	78	Bana et al., 2021
	Mungbean	Fe	87	Ali et al., 2014
**Nutripriming**	Wheat	Zn	12-15	Praharaj et al., 2019
			12	Harris et al., 2008
			900	Johnson et al., 2005
			21-35	Rehman et al., 2015
			900	Johnson et al., 2005
		Mn	30-67	Ullah et al., 2018
			100	Khalid and Malik, 1982
		Fe	70	Sundaria et al., 2019
	Chickpea	Zn	60	Johnson et al., 2005
			29	Harris et al., 2008
	Rice	Zn	580	Johnson et al., 2005
	Lentil	Zn	5	Johnson et al., 2005
	Mungbean	Zn	20	Haider et al., 2020
**Soil less cultivation**	Lettuce	Se	14757	Sahin, 2020
		Zn	28	Sahin, 2020
		Fe	20 - 54	Giordano et al., 2019
	Leafy Brassicas	Zn	52	White et al., 2018
	*B. oleracea*	Zn	687	Barrameda-Medina et al., 2017
	Rice	Fe	51	Chen et al., 2017
	Red Cabbage	Zn	75 to 281	Di et al., 2019
		Fe	278	Di et al., 2019
	Bean	Fe	35	Sida-Arreola et al., 2017
		Zn	75	Sida-Arreola et al., 2017

Fortification levels were calculated based on the increase in content compared to the control.

## 4 Agronomic biofortification in food crops

### 4.1 Agronomic biofortification in cereals

Agronomic micronutrient biofortification proved an alternative strategy to reduce the Fe and Zn deficiency in rice grain (He et al., 2013; Yuan et al., 2013). Fe fertilization has been demonstrated to improve Fe concentration in rice grain when applied as a foliar fertilizer. Studies suggest that adding iron sulfate to germinating rice seeds increased iron concentration in germinated brown rice by 15.6 times as compared to no iron sulfate application (Yuan et al., 2013). Iron application in conjunction with urea fertilizer foliar sprays was found to be positively connected with iron accumulation in wheat grain (Aciksoz et al., 2011). Zinc foliar treatment is a successful agronomic strategy for increasing Zn content and bioavailability in rice grains (Wei et al., 2012; Boonchuay et al., 2013; Mabesa et al., 2013; Ram et al., 2016). When Zn was supplied as a foliar spray coupled with soil application in soils with a lower background level of Zn, the Zn content of rice grain rose (Guo et al., 2016). Foliar Zn has been demonstrated to be useful in lowering anti-nutrient factors such as phytic acid (Yang et al., 2011). Because the use of Zn in combination with NPK fertilizers results in a large improvement in yield, Turkey’s use of Zn-containing NPK fertilizers has climbed from zero in 1994 to 400,000 tonnes per year in the last 10-15 years. In Finland, agronomic Se biofortification in wheat grain has also been found successful (Aro et al., 1995). Zinc nutrition furthers both yield optimization and nutrient enrichment goals. Various Zn fertilizer treatments have been carried out in maize crops (Wang et al., 2012; Zhang et al., 2013; Fahad et al., 2015). An important trace element for human health Se has also been increased in rice grain with the foliar application of selenate (Xu and Hu, 2004; Premarathna et al., 2012; Giacosa et al., 2014; Ros et al., 2016). Selenium (Se) biofortified maize acts as an effective strategy to improve human and animal health (Ros et al., 2016).

Biofertilizers and mycorrhizal fungi, in addition to fertilizers, are widely employed for biofortification (Nooria et al., 2014). For Zn biofortification of wheat grains, *Bacillus aryabhattai* in combination with organic and chemical fertilizers has been found useful (Ramesh et al., 2014; Ramzani et al., 2016). Plant growth promoting rhizobacteria causes enrichment in nutrient content if used as an agronomic approach for the biofortification of staple crops. Sorghum is grown as a grain and fodder crop all over the world. The crop is frequently harmed by nutrient-deficient and polluted soil. Organic and inorganic fertilizers can be used to improve their nutrient profile, as well as yields. Plant growth promoting bacteria and mycorrhizal fungus have proven to have a significant impact on nutrient absorption and metabolic profile (Dhawi et al., 2015; Dhawi et al., 2016). By enhancing the nitrogen and phosphorus content of the soil, *Azospirillum* inoculation with phosphate-solubilizing bacteria boosted grain production and protein content (Patidar and Mali, 2004).

### 4.2 Agronomic biofortification in legumes

Field pea is recognized for their high protein content, and they can be fortified with zinc by applying foliar zinc alone or in combination with soil treatment (Poblaciones et al., 2014). The application of Zn enhanced its concentration in beans with foliar fertilizer application (Ibrahim and Ramadan, 2015; Ram et al., 2016). The uptake of Cu, Mn, and Zn uptake in common beans was stimulated using organic and artificial fertilizers (Ram et al., 2016). Plant growth-promoting actinobacteria have been used to tackle mineral deficiencies in iron, zinc, calcium, manganese, and magnesium (Sathya et al., 2013). Chickpea biofortification mediated by mycorrhizae addressed iron and zinc deficiencies (Pellegrino and Bedini, 2014). Cowpea yield outcomes, nodules plant^-1^, root length, uptake, and nutrient concentration were considerably increased by adding Mo to the soil combined with foliar applications of FeSO_4_-7H_2_O (0.5%) and ZnSO_4_-7H_2_O (0.5%) to address the micronutrient shortage (Dhaliwal et al., 2022). In comparison to the control treatment, foliar application of iron nanoparticles (FeNPs), chelated iron, and sulfate iron fertilizers increased plant height, leaf area, fresh weight, dry weight, the number of branches, the number of pods, and seed weight (CT) in broad-beans (Mahmoud et al., 2022). The overall amount of crude protein, carbohydrates, elements (Fe, Cu, Zn, and Mn), and several amino acids, were all improved by the foliar application of FeNPs to broad-bean seeds. Selenium-enriched soybean has been grown with foliar application of selenium complex fertilizers (Yang et al., 2003). Chickpeas enriched with zinc and selenium were grown by spraying their respective minerals on the leaves (Poblaciones et al., 2014; Shivay et al., 2015).

### 4.3 Agronomic biofortification in oilseeds and vegetables

Canola supplemented with plant growth-promoting rhizobacteria such as *Azospirillum brasilense* and *Azotobacter vinelandii*, as well as fertilizers, had higher levels of oleic acid, linoleic acid, and protein. It was discovered that the presence of rhizobacteria boosted the nutritional content of canola oil significantly (Nosheen et al., 2011). The mustard crop has been mainly targeted for selenium (Se) enhancement; so far Se uptake in the plant has been enhanced using rhizospheric bacteria and their formulations (Yasin et al., 2015b). Zinc concentration increased in both flesh and skin of potatoes using foliar Zn spray; from the experiments, it was concluded that ZnO and ZnSO_4_ were more efficient than ZnNO_3_ in increasing Zn concentration while maintaining yields (White et al., 2017). With foliar treatment of selenite and selenate, the potato’s selenium concentration increased (Poggi et al., 2000; Cuderman et al., 2008). When treated with their respective fertilizers, the biofortification of iron and iodine has been recorded in tomato crops (Landini et al., 2011). Martin et al., 2020 conducted a study on the soil and foliar application of zinc to biofortify broccoli (2020). The results revealed that broccoli acquired more zinc when both topically and subsurfacely given zinc sulfate. Foliar Fe application can be an efficient agronomic technique for producing Fe-biofortified quinoa grains, as described by Lata-tenesaca et al. (2023). Due to the plant’s sensitivity to Zn, according to De Moraes et al., 2022, it is necessary to regulate Zn concentrations for agronomic biofortification during each growing season to maintain optimal production and quality. To raise the selenium concentration in tomato plants and fruits, Rahim et al. (2020) studied the agronomic biofortification of tomatoes by applying sodium selenite at several doses (Na_2_SeO_3_). When sodium selenite (5 mg L^-1^) was given to various plant parts and fruits, the most agronomic factors and selenium content were observed to improve. Broccoli and carrots were bio-fortified by foliar application of a solution of Se that was enriched with Se content (Banuelos et al., 2015).

## 5 Application techniques in agronomic biofortification

Several types of agronomic biofortification techniques have been tested for effectiveness worldwide (**Table 1**). Of many, soil application of micronutrient fertilizer for plants to take up nutrients, foliar application using diluted fertilizer sprays, nutripriming, and soilless cultivation are the major techniques.

### 5.1 Soil application

Soil application of micronutrients helps in replenishing the micronutrients in the soil on which a crop or plant is grown. This is a conventionally used technique. A higher application of micronutrients is recommended for crops that are quite sensitive to micronutrient deficiency (Martens and Westermann, 1991). Soil application of micronutrients is a less efficient method of fertilizer application and increases the cost of production (Savithri et al., 1999). The banding placement requires three times less micronutrient fertilizer as compared to broadcasting (Sarwar et al., 2017). Soil Zn fertilization may increase the yield of the crop but is comparatively less effective in increasing Zn content in grain as well as it has low fertilizer use efficiency (Singh and Mann, 2007; Chattha et al., 2017). To address the micronutrient deficiency, adding Mo to the soil along with foliar treatments of FeSO_4_-7H_2_O (0.5%) and ZnSO_4_-7H_2_O (0.5%) significantly boosted cowpea production outcome, nodules plant^-1^, root length, absorption, and nutrient concentration (Dhaliwal et al., 2022). It is claimed that soil application along with the foliar application is more effective and better to increase grain production compared to soil or foliar application alone. Several workers have demonstrated successful biofortification using soil application of micronutrient fertilizers (**Table 1**). Though soil application is the most common method of micronutrient application to crops, it has mostly been tested for crop productivity improvement rather than biofortification. This method has low micronutrient use efficiency, less cost-effectiveness, and pollutes soil over time due to excessive buildup of unused micronutrients.

### 5.2 Foliar application

Foliar application is a better option than soil application as the loss of micronutrients is very less in this mode of application and the micronutrients are directly adsorbed by the plant tissue (Johnson et al., 2005). Zou et al. (2012) found that foliar feeding of Zn was superior in increasing grain Zn content. Foliar application at a later stage is more beneficial for grain biofortification than foliar application at the early vegetative stages (Yilmaz et al., 1997). Foliar Zn application after flowering, and during the early milk and dough stages, boosted grain Zn content more than other earlier applications, according to Phattarakul et al. (2012). Foliar Zn spraying improved test weight and grain protein content in alkaline soils without impacting biological yield (Khattak et al., 2015). During anthesis, foliar spraying of FeSO_4_ enhanced grain protein content and gluten content in durum wheat, especially at a seed rate of 125 kg ha^-1^ (Melash et al., 2016). Foliar treatment is recognized as an important approach for addressing micronutrient deficiency in crops in arid and semi-arid climates as there is less availability of water for irrigation and solubilization of soil-applied fertilizer (Chapagain and Wiesman, 2004). Foliar Zn application increased grain Zn and Fe concentrations by 99% and 8%, respectively, while foliar Mn application increased grain Mn content by 7% (Pahlavan-Rad and Pessarakli, 2009). According to Narwal et al. (2012), foliar Zn, Fe, and Mn treatment improved the level of these nutrients in 14 winter wheat types. Since soil application has the disadvantage of the fixation of micronutrients in alkaline and calcareous soils (Alloway, 2008). The foliar application makes better sense under such conditions. Zhang et al. (2012) stated that as foliar Zn treatment was more successful than soil Zn application in enriching wheat grain with Zn, it constituted an effective strategy to give more dietary Zn from goods derived from biofortified wheat to people. Foliar Zn application decreased the molar ratio of phytic acid to Zn while also increasing the Zn concentration in flour. Foliar feeding of micronutrients appreciably contributes to the biofortification of the wheat crop (Cakmak, 2008; Cakmak, 2010). Nandita et al. (2022) revealed that three foliar sprays of Zn @ 0.5% + Fe @ 0.2% + B @ 0.3% + Cu @ 0.1% from May to July can be advised to get the highest production with enhanced fruit quality of Mosambi orchard. The application of nano-iron to soybean foliage boosted yield, seed quality, and drought tolerance (Dola et al., 2022). Foliar application is the most adopted technique for micronutrient biofortification as it is simple to adopt, more fertilizer use efficient, requires less infrastructure, and does not require technical knowhow that may be needed for techniques like nutripriming and soil-less cultivation which are discussed in proceeding sections.

### 5.3 Nutripriming

Nutripriming or seed-priming is the soaking of seeds before planting in a solution containing nutrients (Lutts et al., 2016; Farooq et al., 2019; Raj and Raj, 2019). Seed-priming has been primarily used to enhance germination, root system development, seedling establishment, and yield improvement (Lutts et al., 2016; Farooq et al., 2019; Raj and Raj, 2019). However, some researchers have also noted improved grain nutrient content with the use of nutripriming. Zinc-nutripriming with ZnSO_4_ (0.4%) improved grain Zn content by 29% in chickpea (Harris et al., 2008), and 12% to 15% in wheat (Harris et al., 2008; Praharaj et al., 2019). An additional benefit of seed priming is that farmers can adopt this approach without any added cost as the micronutrients are added to seeds before sowing (Harris et al., 2008). Micronutrient seed priming is cost-effective, environmentally friendly, and results in improved micronutrient content and crop yield. Seed priming has rarely been found to be ineffective (Farooq et al., 2012). In field trials, magneto-priming of seeds relieved salt stress and improved seedling characteristics in barley plants at the early seedling stage (Cheikh et al., 2018). Zn content of grains increased from 21 to 35 percent after nutripriming with ZnSO_4_ and ZnCl_2_, respectively at a rate of 1.25 g Zn kg^-1^ seed, and grain production increased by 33-55 percent (Rehman and Farooq, 2016). All kinds of seed priming, including hydro-priming, promote seed germination, according to Choukri et al. (2022). With the use of zinc priming, the seeds were enriched with this element, and it also improved grain yield. Specifically, seedlings treated for 24 hours with 0.5% Zn sulfate had a 47% increase in yield and a 15% increase in Zn content. Nano-priming is substantially more efficient than any other seed priming method. The development of increased surface response and electron exchange capacities connected to various parts of plant cells and tissues are two of nanoparticles’ (NPs) significant properties in seed priming. Nano-priming also results in the generation of hydroxyl radicals, which act as an inducer for the quick breakdown of starch, reactive oxygen species (ROS), and antioxidant mechanisms in seeds, in addition to the production of nanopores in the shoot that aid in water absorption. Additionally, it promotes the activation of aquaporin genes, which are important for water intake, and the transport of H2O2, or ROS, across biological membranes. Nano-priming increases starch breakdown by activating amylase, which in turn promotes seed germination (Nile et al., 2022). Selenium priming may minimize the harmful effects of drought stress by altering the germination and metabolic properties of quinoa (Gholami et al., 2022). The effectiveness of seed priming largely depends on several factors like genotype, crop type, duration of the nutrient priming, osmotic potential of priming solution, and environmental conditions (Farooq et al., 2019; Raj and Raj, 2019; Waqas et al., 2019). Besides nutripriming techniques are not very well known to farmers as these involve technical aspects of priming methodology. Priming may reduce shelf life, and therefore, the seeds would need either ideal storage or immediate use/sowing (Murphy, 2017).

### 5.4 Soilless cultivation

Soilless cultivation is a more recent method of crop production that utilizes inert organic, inorganic, or liquid growing media with desired concentration and form of nutrients. There are various soilless systems, including hydroponic, aeroponic, vertical farming, and others, depending on the needs and type of crop. Soilless cultivation of food crops is making its place in human nutrition, especially the role of microgreens where micronutrients are supplemented through micronutrient-rich media (Rouphael and Kyriacou, 2018). In soilless cultivation, plant productivity can be optimized more efficiently with strict regulation of environmental conditions viz. temperature, and light along with the nutrient concentration in solutions (Gruda, 2005; Gruda, 2009) resulting in the maximization of root contact with nutrient supply (Treftz and Omaye, 2016). Continuous root contact with fertilizer solution enhances nutrient uptake, translocation, and accumulation, ensuring consistent results for nutritional quality (Wiesner-Reinhold et al., 2017; Rouphael and Kyriacou, 2018). Furthermore, soilless cultivation extends the cultivation cycle and allows year-round production while ignoring soil restrictions like soil fertility and disease transmission (Tomasi et al., 2015; Rouphael and Kyriacou, 2018). Other benefits of soilless agriculture include the absence of weeds, the lack of a high labor need, easy harvesting and processing, and an automated system for plant maintenance (Tomasi et al., 2015). Enough evidence exists that claims that specific micronutrient media in soilless cultivation results in higher plant micronutrient content. Soilless cultivation has been found to successfully increase Zn, and selenium in lettuce (*Lactuca sativa* L.) (Sahin, 2020), and Zn content in white cabbage (*Brassica oleracea* L.) (Barrameda-Medina et al., 2017). The role of soilless growth microgreens in combating micronutrient deficiency is realized by people worldwide in recent years probably because of high micronutrient content along with flavor-enhancing properties, soilless cultivation (Xiao et al., 2012; Kyriacou et al., 2016). Brassicaceae microgreens have been soilless biofortified with Zn and Fe, coriander (*Coriandrum sativum* L.) and tatsoi (*Brassica rapa* subsp. narinosa) have been biofortified successfully with selenium (Puccinelli et al., 2019; Pannico et al., 2020). Cherry tomatoes can be effectively biofortified with Fe to treat its deficiency and improve fruit quality (Buturi et al., 2022). By cultivating cucumbers without losing any nutrient solution, it is possible to save money on energy used for disinfection, use less water and fertilizer, and cause less environmental damage (Ding et al., 2022). Soilless cultivation needs infrastructure development that would be out of reach for many regions, though it is environment-friendly and highly efficient.

## 6 Advances in fertilizer forms revive agronomic biofortification

**Figure 3:**
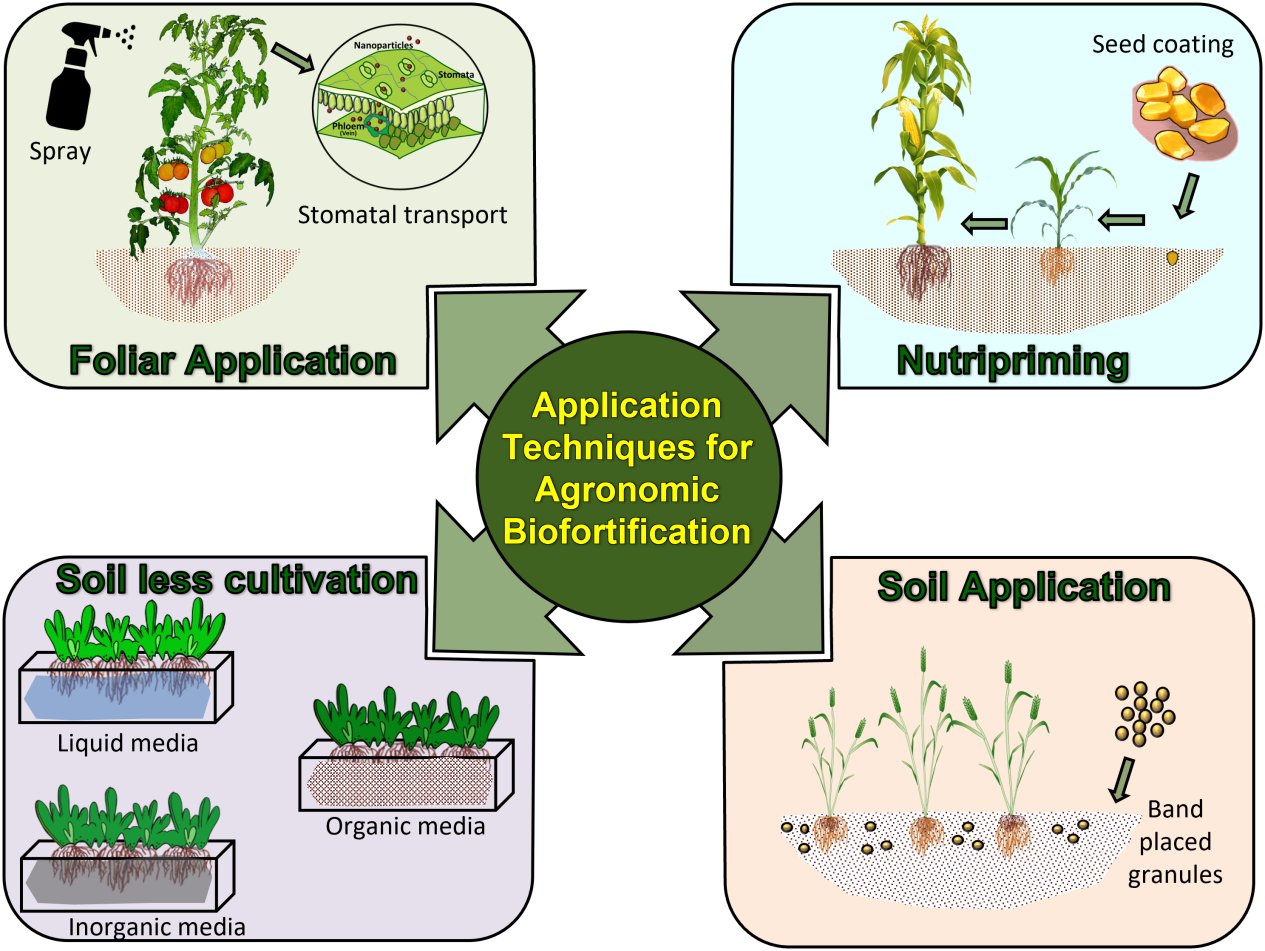
Application techniques used for agronomic biofortification of crops.

Inorganic fertilizers are required to supplement the nutrient requirement of crops whether it is through soil application, foliar application, or by any other suitable method (**Figure 3**). Due to its frequent usage in agriculture and lack of recycling after crop harvest, the soil can get low in some nutrients. Most conventional fertilizers contain only macronutrients. A typical fertilizer with all the nutrients should have a balanced proportion of N (2-4 percent), P (0.3-1 percent), K (1.5-5 percent), S (0.15-0.8 percent), Ca (0.2-1.5 percent), Mg (0.15-1 percent), Zn (10-100 ppm), Fe (20-00 ppm), Mn (15-250 ppm), Cl (4-50 ppm), Co (2.5-50 ppm), Cu (5-75 ppm), and Mo (0.03-10 ppm) (McKenzie, 1998). The most prevalent method of agronomically biofortifying crops is to use inorganic fertilizers. The use of micronutrient fertilizers has been found to enhance micronutrient content in different agroecosystems (Hirschi, 2009; Zou et al., 2012; Garg et al., 2018). Agronomic Biofortification with different micronutrients can be done using their popular commercial formulations such as ZnSO_4_, FeSO_4_, CuSO_4,_ and MnSO_4_. The combined application of Zn and Fe leads to increased grain Zn, Fe, crude fiber, and protein content, whereas the application of Fe fertilizer alone improves grain Fe content (Niyigaba et al., 2019). Soil and foliar fertilization increased Zn content in corn (Zea mays L.) (Zhang et al., 2013; Fahad et al., 2015; Maqbool and Beshir, 2019), wheat (*Triticum aestivum* L.) (Zou et al., 2012; Wang et al., 2012), peas (Pisum sativum L.) (Poblaciones and Rengel, 2016), chickpeas (Shivay et al., 2016b), potatoes (*Solanum tuberosum* L.) (White et al., 2017; Kromann et al., 2017), and rice (Boonchuay et al., 2013; Guo et al., 2016; Ram et al., 2016; Nakandalage et al., 2016). Although the use of inorganic fertilizers is a cost-effective, simple, and quick method of agronomic biofortification, it has several drawbacks. The main downside is related to the misuse of inorganic fertilizers, which has negative environmental consequences such as water pollution, algal bloom, and biodiversity loss in various natural systems (Zhang et al., 2019; Schier et al., 2019; Jewell et al., 2020). Moreover, these are expensive, if considered for bulk application to soil, and labor-intensive to apply, which may further impoverish farmers with small holdings. Besides this, scheduling fertilizer applications to get the best biofortification, as well as economic benefits, is another challenge as it is crop-dependent (Phattarakul et al., 2012; Mabesa et al., 2013; Rodrigo et al., 2014). Advances have been made in the fertilizer forms in terms of nutrient form, content, particle sizes, and complexion of nutrients to enhance the efficiency of fertilizers for biofortification (**Table 2**). These advanced forms are discussed in the following sections.

**Table 2:** Advanced fertilizer forms and techniques that have been successfully used for major mineral micronutrients in different crops.

Fortification technique	Type	Crop Biofortified	References
**Mineral fertilizers**	Zn	Rice, Wheat, Corn, Finger Millet, Peas, Chickpea, Potato, Cowpea	Guo et al., 2016; Yamunarani et al., 2016; Poblaciones and Rengel, 2016; Shivay et al., 2016a; White et al., 2017; Maqbool and Beshir., 2019; López-Morales et al., 2020
	Fe	Rice, Wheat, Corn, Finger Millet, Peas, Chickpea, Cowpea	Kabir et al., 2016; Aziz et al., 2019; Pal et al., 2019; Prom-U-Thai et al., 2020; López-Morales et al., 2020; Grujcic et al., 2021; Khobragade et al., 2022
	Cu	Eggplant, Wheat, Corn, Finger Millet, Cowpea	Tuhy et al., 2015; Yamunarani et al., 2016; Aziz et al., 2019; López-Morales et al., 2020; Bana et al., 2021
	Mn	Wheat, Corn, Finger millet; Cowpea,	Tuhy et al., 2015; Klikocka and Marks, 2018; López-Morales et al., 2020
**Biofertilizers**	Zn	Corn, Wheat, Barley, Soybean, Parsley, Mentha	Ramesh et al., 2014; Ramesh et al., 2014; Prasanna et al., 2015; Coccina et al., 2019; Gashgari et al., 2020; Gashgari et al., 2020
	Fe	Corn, Parsley, Mentha, Mungbean	Sharma and Johri, 2003; Patel et al., 2018; Gashgari et al., 2020
	Mn	Parsley, Mentha	Gashgari et al., 2020
	Cu	parsley, Mentha	Gashgari et al., 2020
	Se	Wheat, Shallot, Chickpea	Yasin et al., 2015a; Golubkina et al., 2019
**Chelates**	Zn	Unhusked Rice, Chickpea, Rice, Wheat, Pear,	Shivay and Prasad, 2012; Shivay et al., 2015; Koksal et al., 1999; Zhao et al., 2014
	Fe	Wheat, Soybean, Cowpea, Pear	Koksal et al., 1999; Ghasemi et al., 2013; Márquez-Quiroz et al., 2015; Sharma et al., 2019
	Mn	Cowpea, Pear	Koksal et al., 1999; Márquez-Quiroz et al., 2015
	Cu	Pear, Wheat	Modaihsh, 1997; Koksal et al., 1999
**Nanofertilizers**	Zn	Okra, Wheat, Corn, Maize, Coffee,	Yahyaoui et al., 2017; Deshpande et al., 2017; Wang and Nguyen, 2018; Choudhary et al., 2019; Tarafder et al., 2020
	Fe	Okra, Corn, Soybean, Wheat	Li et al., 2016; Sharma et al., 2019; Al-Juthery et al., 2019; Tarafder et al., 2020
	Cu	Okra, Maize, Wheat	Al-Juthery et al., 2019; Tarafder et al., 2020; Sharma et al., 2020
	Mn	Wheat, Lettuce	Liu et al., 2016; Al-Juthery et al., 2019
	Se	Garlic, Groundnut	Li et al., 2020; Hussein et al., 2019
**Integrated nutrient management**	Zn	Wheat, Cauliflower, Maize, Peanut	Prasad et al., 2012; Yadav et al., 2014; Tariq et al., 2014; Klikocka and Marks, 2018
	Fe	Wheat, Soybean, Sugarcane (stalk)	Caliskan et al., 2008; Mishra et al., 2014; Klikocka and Marks, 2018
	Mn	Wheat, Tomato, Sugarcane (stalk)	Kleiber, 2014; Mishra et al., 2014; Klikocka and Marks, 2018
	Cu	Wheat, Spinach	Obrador et al., 2013; Klikocka and Marks, 2018

### 6.1 Biofertilizers

**Figure 4:**
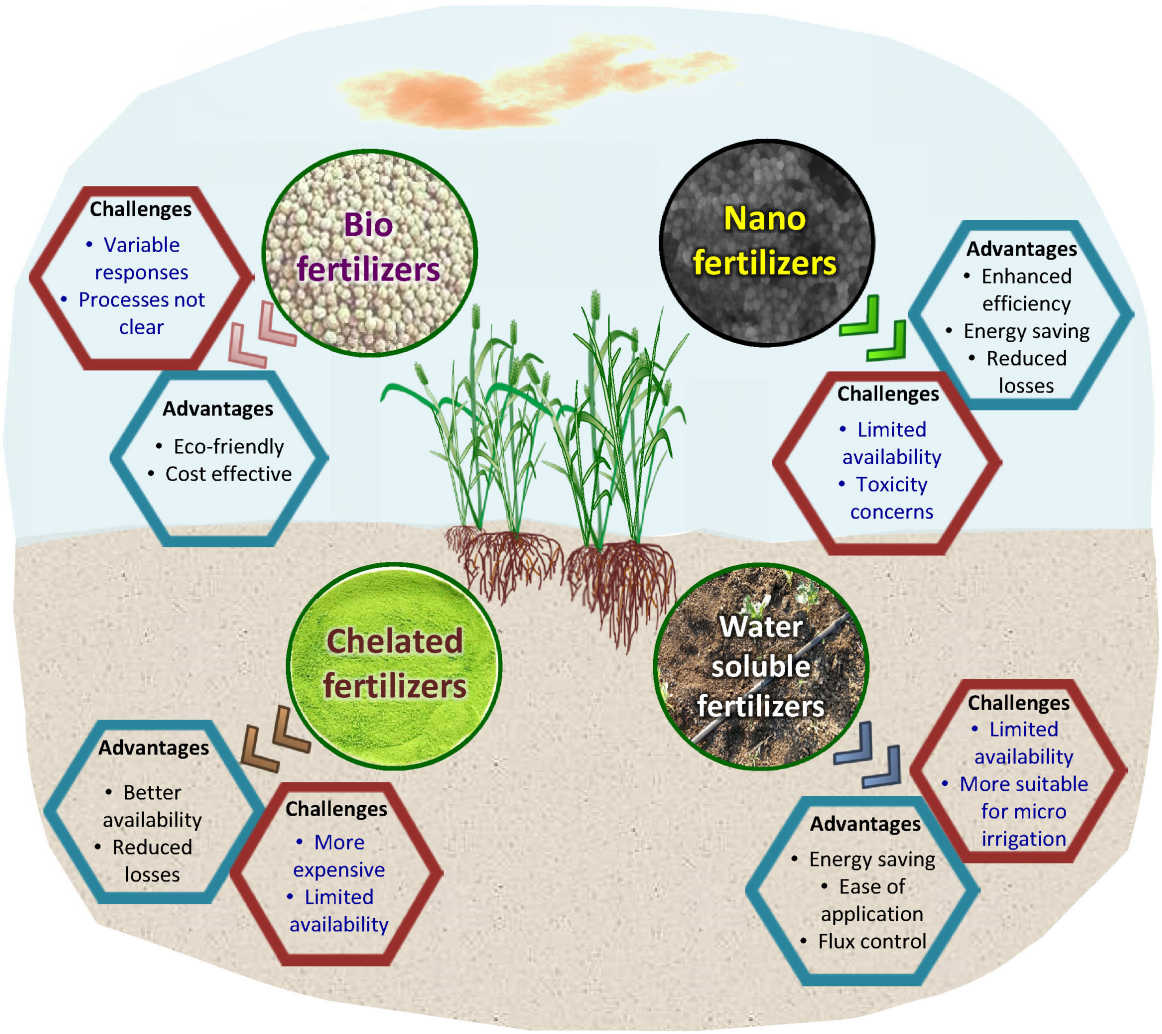
Advancements in fertilizer forms to increase the efficiency and relevance of agronomic biofortification.

Biofertilizers are microbial inoculant preparations consisting of microorganisms that help in improving the growth and productivity of the host plant (Sahoo et al., 2013; Bhardwaj et al., 2014). These are generally referred to as plant growth-promoting microorganisms. Biofertilizers are useful since they are inexpensive and simple to make, as well as being sustainable in agriculture and freely accessible. These bacteria increase the supply and availability of nutrients, hence increasing nutrient content (Sahoo et al., 2013; Bhardwaj et al., 2014). Zn biofortification in corn is facilitated by cyanobacteria (*Azotobacter* sp. and *Anabaena* sp.) and *Bacillus aryabhattai* (Prasanna et al., 2015), wheat (Ramesh et al., 2014), and soybeans (*Glycine max* L.) (Ramesh et al., 2014), respectively. Microbial intervention is suggested as a means that can be used to eliminate Zn deficiency (Dotaniya et al., 2016). Arbuscular mycorrhiza fungi have been found to enhance root development and ensure uptake of P, N, Zn, Cu, Mn, and Fe. *Rhizophagus irregularis* increases the primary metabolites and minerals like Fe, Mn, Cu, and Zn in medicinal plants such as *Mentha pulegium* and *Petroselinum Hortense* (Gashgari et al., 2020). Pseudomonas spp. and *Pseudomonas chlororaphis* isolated from maize improved Fe uptake, germination, plant growth, and crop output, according to Sharma and Johri, (2003). Ferric forms of iron (Fe^3+^) have very low solubility and cannot be taken up by plants, whereas microorganisms secrete Fe-chelating compounds called siderophores, which facilitate the uptake of microelements at different pH ranges. *Rhizophagus irregularis* and arbuscular mycorrhizal fungi promote the uptake of soil-applied Zn, mobilizing micronutrients in wheat and barley, according to Coccina et al. (2019). Despite the practical promises, there remain some roadblocks. Major ones are the identification of proper plant growth-promoting microorganisms for each host crop, and biofertilizers’ short life due to variation in the different agro-ecosystems environments (**Figure 4**). Improper storage and application of biofertilizers may not result in the biofortification benefits achieved under ideal test conditions.

### 6.2 Nanofertilizers

Nano fertilizers are the fertilizer forms in which the active ingredients are in the size range of 1- 100 nm particles/micelles/pockets, dispersed, adsorbed, entrapped, or encapsulated in a host material (Bhardwaj et al., 2022). Wheat crop nanobiofortification has gained popularity as a successful alternative method to improve nutrition (Khan et al., 2021). Nanofertilizers are tailored fertilizers with the possibility to revolutionize the current agricultural system (Dimkpa and bindraban, 2016; Manjunatha et al., 2016; Rai et al., 2018). Nanofertilizers are elegant delivery structures that are safe, target-bound, and easy to apply. Because of the high surface area to volume ratio, most polymeric-type fertilizers make nanoformulations more effective, slow-release, and efficient nutrient suppliers to crops. Thus nano-fertilization serves as a platform for a sustainable and novel nutrient delivery system that can explore the nanoporous surface of a plant. The nanofertilizers may include zinc oxide nanoparticles, silica, iron, and titanium dioxide as well as core-shell quantum dots (QDs) of Zn, Fe, Mn, Cu, and Ti (Prasad et al., 2017; Bhardwaj et al., 2022). The success of nanofertilizers depends on different factors viz. plant species and chemical properties such as size, concentration, and composition of nanomaterials (Thakur et al., 2018). As nanofertilizers are engineered in such a way that they could address the deficiency of a particular nutrient, fortification through nano-nutrients seems to be an interesting option. With the use of these fertilizers, the plant will not only grow but will also accumulate such nutrients in its consumable parts (Li et al., 2016). Dapkekar et al. (2018) reported that when two durum wheat varieties (MACS 3125 and UC 1114) were treated with zinc complexed chitosan nanoparticles (Zn-CNP (40 mg L^-1^)) and conventionally applied ZnSO_4_ (0.2%; 400 mg L^−1^ zinc) grain Zn enrichment was observed to increase by ~36% with Zn-CNP nano-carrier and ~50% with ZnSO_4_ even though 10-fold lower concentration of zinc complexed chitosan nanoparticles was used. The use of nanofertilizers enhanced grain Zn content, protein content, as well as test weight of durum wheat varieties.

According to Hussain et al. (2021), foliar exposure of wheat plants to ZnO nanofertilizer increased Zn content in various parts of the plant; a foliar treatment of 100 mg L^-1^ of ZnO nanofertilizer resulted in Zn concentrations of 100–150 mg kg^-1^ dry weight in root and shoot tissues and 45 mg kg^-1^ in wheat grain. The use of chitosan-complexed Zn nanofertilizers increased grain Zn content by around 21–27g g^-1^, and foliar application of ZnO nanofertilizer revealed grain Zn accumulation in distinct seed sections (aleurone layer and embryo) that was similar to soil uptake (Doolette et al., 2020). Soil application of Fe nanofertilizer showed higher shoot Fe concentrations than that through foliar spray but grain Fe content was found to be greater (110 mg kg^-1^) with the foliar application as compared to the soil application (90 mg kg^-1^) (Hussain et al., 2019). Alidoust and Isoda (2013) found that when citrate-coated nano Fe_2_O_3_ and Fe_2_O_3_ (conventional form) were applied to soybean plants, no phytotoxic effects were observed. Nano-Fe_2_O_3_ had a more stimulating effect on root growth than conventional Fe_2_O_3_. Using *Vigna radiata* as a test crop, Pradhan et al. (2013) explored the potential of nano-Mn as a manganese sulfate (MnSO_4_) alternative. In addition to fundamental observations, it was discovered that when nano-Mn was employed as a source of fertilizer, Mn accumulation in seeds increased. Dimkpa et al. (2018) carried out a study on the wheat plant using nano-Mn, bulk, and ionic form of Mn as sources of nutrients and it was found that the use of nano-Mn resulted in more plant growth, grain yield, and nutrient acquisition in comparison to the bulk and ionic form of Mn. Grain Mn translocation efficiency was also found higher in plants treated with nano-Mn.

### 6.3 Chelated fertilizers

Chelated fertilizers are those fertilizer forms in which the nutrient ion is encircled by a macro-sized organic molecule (Ligand/Chelator) which protects from precipitation, immobilization, and oxidation. Chelated fertilizers have been shown to have better protection of nutrients from the soil conditions (pH, moisture, etc.) that cause immobilization or loss of nutrients *via* oxidation, precipitation, or leaching. Therefore, chelated nutrients have reduced losses and higher uptake by plants. Chelated micronutrients are more efficient than inorganic micronutrient fertilizers, and a high percentage of nutrients further makes them superior to organic and bio-fertilizers. Chelated nutrients have reduced environmental loss. Zhao et al. (2019) found that Zn-EDTA fertilization achieved greater Zn biofortification than ZnSO_4_.7H_2_O fertilization, even with a lower treatment volume, in a greenhouse study. Zinc content in wheat was increased by foliar application of Zn-containing salts and Zn chelates (e.g., ZnSO_4_, Zinc-EDTA, generally @ 0.5-0.7 kg/ha) (Kutman et al., 2010; Zhao et al., 2014). Lycine chelate, when applied to Cd-contaminated soils, produced Zn enrichment in wheat while lowering Cd levels in the plant (Rizwan et al., 2017). Zn(Gly)2 alone or with nitrogenous fertilizers improved Zn and Fe content in wheat grain and flour. Ghasemi et al. (2013) observed that Zn-Amino Acid complexes (ZnAAC) increased the efficacy of Zn uptake by lettuce cultivars, compared to ZnSO4. ZnAAC had a stimulating effect on the root and shoot growth resulting in better yields as well as Zn uptake. Sometimes bioremediation can also be used as a strategy for biofortification as observed by Bilski et al. (2012). Increased crop development was observed when six crop plants, including wheat and barley, were cultivated on coal fly ash naturally enriched with micronutrients like Fe, Zn, and Se. When both were applied at 0.5 percent concentration, Shivay et al. (2016a) found that three foliar applications of Zn-EDTA (at tillering, booting, and grain filling stages) resulted in significantly better growth, higher values for yield attributes, high grain and straw yield, higher concentration, and high uptake of Zn than ZnSO_4_.H_2_O.

## 7 The way forward and future course

**Figure 5:**
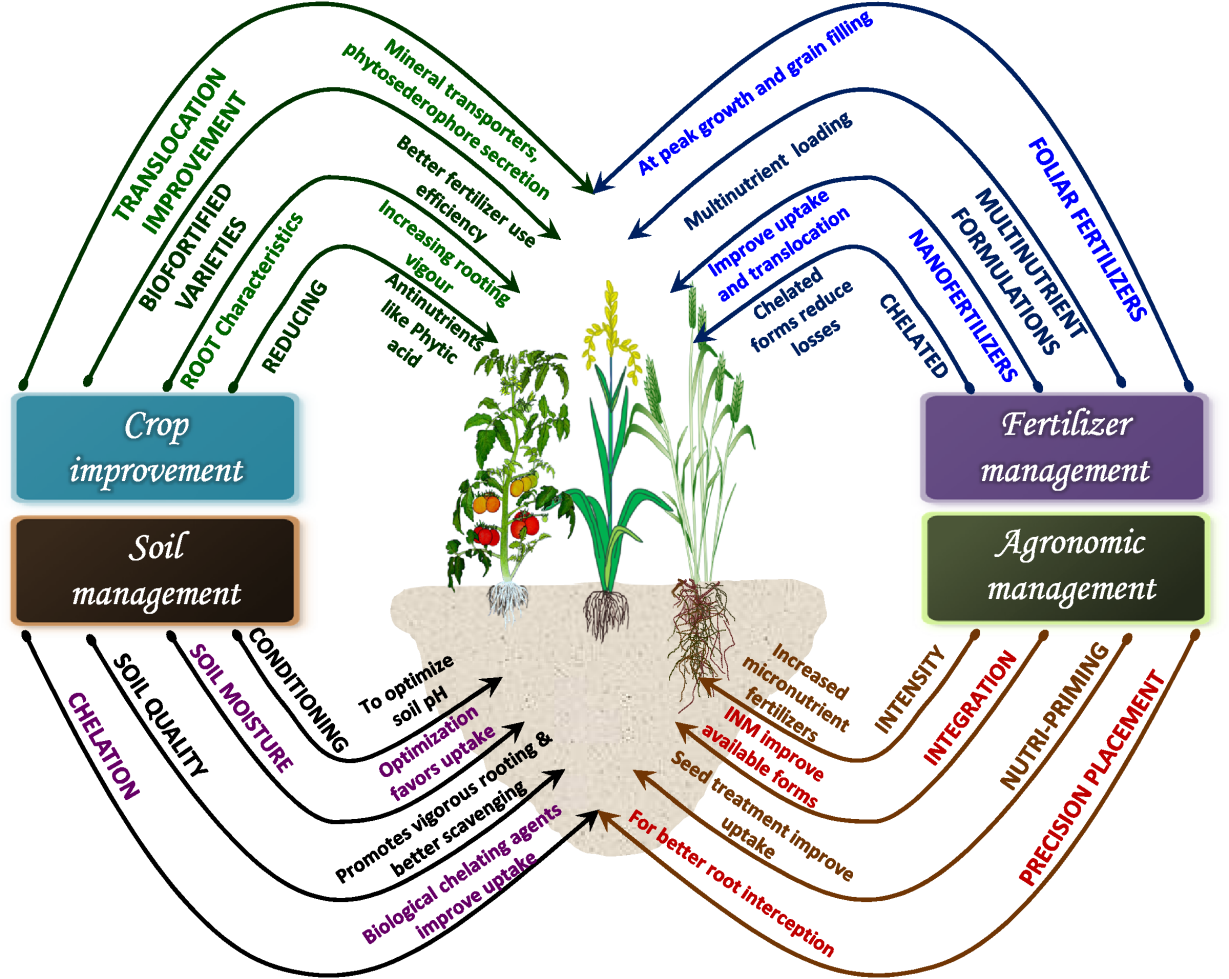
Agronomic, soil, fertilizer, and crop management based interventions to enhance the success of agronomic biofortification.

Agronomic biofortification of food crops has been recently overshadowed by genetic and transgenic methods of biofortification. This recent focus on biological fortification by improving the capacity of plants to naturally assimilate mineral micronutrients in tissues (genetic and transgenic biofortification) is primarily because of poor nutrient use efficiency and fortification potential noted for agronomic biofortification *via* conventionally used soil-application based techniques. The conventional soil application of mineral micronutrients was initially focused on improvement in crop yield rather than improving nutrient status in consumable parts, success has been achieved in that regard. The location specificity (soil and site conditions) and fertilizer characteristics have more to do with the poor nutrient use efficiency and biofortification potential than the agronomic biofortification technique as such. Poor micronutrient use efficiency of crops and as a result, high fertilizer doses not only increase the level of nutrients to the status of a pollutant in soil but it also incurs substantial financial losses to a farmer, discouraging him/her from future investment in it. The buildup of nutrients in the soil due to fertilization has been noted in agricultural soils of some regions without much transport to consumable parts. Agronomic biofortification is simple to follow and therefore can be easily adopted by growers. Since agronomic biofortification does not directly benefit in terms of crop yield, in many cases, farmers/growers tend to not care for it as it does translate into direct economic benefits. On the other hand, if a crop variety is developed to naturally take up more mineral micronutrients from soil (genetic biofortification) then the fortification of that micronutrient need not depend on the grower to apply that micronutrient, which is a general assumption. Yet, under such conditions as well, agronomic biofortification would play an important role. A genetically biofortified crop variety would scavenge the micronutrients from soil which is also a limited and exhaustible pool. If not immediately, after some years growing genetically biofortified varieties would need to be complemented by agronomic biofortification to sustain source-sink linkage. The effectiveness of agronomic biofortification has improved in recent times with the invention of several types of specialty fertilizers like nano-fertilizers, chelated fertilizers, biofertilizers, and water-soluble fertilizers that have higher efficiency of nutrient use by plants, and better nutrient translocation to consumable plant parts (**Figure 5**). Several new agronomic biofortification techniques like foliar application, nutripriming, soilless culture, and precision application in soil, etc. have further increased the relevance of agronomic biofortification. An integrated effort *via* varietal improvement for increased translocation of micronutrients to consumable parts of plants, improved soil conditioning by farmers for optimizing nutrient uptake by plants combined with the advancements in agronomic biofortification techniques can make agronomic biofortification highly relevant in times to come. It can become an important strategy to combat malnutrition and global food insecurity.

## 8 Conclusion

The global health crisis witnessed under the Corona Pandemic has turned the focus of the global population toward the nutritional quality of food, especially micronutrients that play a crucial role in developing body immunity. During this time, mineral micronutrient supplementation peaked, and the value of biofortification during crop production became widely recognized. Diversity in food products can help the fight against micronutrient malnutrition, but not everyone can afford it, particularly in emerging and underdeveloped nations. Many studies have shown the effectiveness of agronomic biofortification in enriching plants and their consumable parts with intended micronutrients that can be useful in combating malnutrition globally. In general, increasing the concentration of vital nutrients in cereals, vegetables, fruits, and other local foods would help combat the adverse effects of climate change or any other global crisis (economic or pandemic induced) *via* the availability of lesser yet richer food. Intensifying agronomic biofortification of crops can act as an important strategy. Important advances have been made in fertilizer formulation technology as well as application methodology that has enhanced the effectiveness of agronomic biofortification. Amongst the new fertilizer forms, nano-fertilizers, chelated fertilizers, and biofertilizers are the most rapidly advancing ones. These fertilizer forms have been widely reported to increase micronutrient biofortification, and provide better micronutrient use efficiency. Foliar application (spray of water-soluble forms), nutripriming of seeds, and soil-less cultivation (as in hydroponics and aeroponics) enhance micronutrient use efficiency manifold compared to soil application while the biofortification levels achieved using these techniques are also significant. Amongst the three techniques, the foliar application has been gaining popularity due to simple operations while nutripriming, and soilless cultivation are rapidly gaining ground, especially in urban and hard-to-farms regions. The latter two techniques, though more efficient, may need farmers to get technical knowledge for application and achieve desirable results. Standardization of methodology and development of packages of best practices would help the adoption of these technologies and micronutrient biofortification worldwide. The advances in these technologies have reinforced the importance of agronomic biofortification for micronutrient enrichment. Investing in the advancement of agronomic biofortification techniques even supports the success of genetic and transgenic biofortification as the supply of available forms of micronutrients to micronutrient-hungry genetically-biofortified crops can only be ensured using agronomic biofortification. To assure micronutrient enrichment of crops and fight hidden hunger, agronomic biofortification should be a key area of future focus.

## Data availability statement

The original contributions presented in the study are included in the article/supplementary material. Further inquiries can be directed to the corresponding author.

## Ethics statement

Written informed consent was obtained from the individuals/minor(s)' legal guardians for the publication of any potentially identifiable images or data included in this article.

## Author contributions

AB conceptualized the study, wrote the original draft, and prepared visualizations. SC wrote the original draft and prepared tables. KM wrote the original draft, reviewed it, and edited it. RK wrote, reviewed, and edited. AK wrote, reviewed, and edited. RY reviewed and edited. All authors contributed to the article and approved the submitted version.
